# Is Lhasa Tibetan Sign Language emerging, endangered, or both?

**DOI:** 10.1515/ijsl-2017-0005

**Published:** 2017-05-02

**Authors:** Theresia Hofer

**Keywords:** Tibetan Sign Language (TibSL), deaf Lhasa Tibetans, sign language vitality and endangerment assessment, Tibet Deaf Association (TDA), Tibet Autonomous Region (TAR), China

## Abstract

This article offers the first overview of the recent emergence of Tibetan Sign Language (TibSL) in Lhasa, capital of the Tibet Autonomous Region (TAR), China. Drawing on short anthropological fieldwork, in 2007 and 2014, with people and organisations involved in the formalisation and promotion of TibSL, the author discusses her findings within the nine-fold UNESCO model for assessing linguistic vitality and endangerment. She follows the adaptation of this model to assess signed languages by the Institute of Sign Languages and Deaf Studies (iSLanDS) at the University of Central Lancashire. The appraisal shows that TibSL appears to be between “severely” and “definitely” endangered, adding to the extant studies on the widespread phenomenon of sign language endangerment. Possible future influences and developments regarding the vitality and use of TibSL in Central Tibet and across the Tibetan plateau are then discussed and certain additions, not considered within the existing assessment model, suggested. In concluding, the article places the situation of TibSL within the wider circumstances of minority (sign) languages in China, Chinese Sign Language (CSL), and the post-2008 movement to promote and use “pure Tibetan language”.

## 1 Introduction

One afternoon in the early summer of 2007, my friend Sonam and I rode on a busy public bus out of downtown Lhasa. As we looked out, passing concrete two-storey houses with shops on the ground floor, their signs featuring large Chinese and comparatively tiny Tibetan letters, I heard taxis and motorcyclists blowing their horns. Lhamo signed to me that she would prefer that the Chinese leave Tibet. Although hard of hearing, and despite her spoken Tibetan being easy for me to understand, she continued to sign, adding that she liked foreigners and we were welcome to stay. I was a little perplexed, and ever since have been pondering what an apparent “disability” may mean for the possibilities of circumventing constraints commonly experienced among Tibetans under the current political circumstances. Or was her choice to sign here simply to teach me more Tibetan Sign Language? Lhasa is a city fraught with countless political sensitivities and its Tibetan inhabitants tend to be careful about what they say and to whom. Like anywhere in the world, the signs that deaf people use are linked to local political, historical and social circumstances. In TibSL, the sign for a Chinese person, for instance, is one’s hand forming a shield in front of the head, presumably indicating the caps worn by state police and military personnel, ever present in the city since the arrival of People’s Liberation Army (PLA) troops in 1951. The sign for foreigner, on the other hand, seemed relatively benign, the fist placed in front of the nose in reference to Europeans’ typically longer noses.

Over the past 15 years, significant transformations have been taking place in the lives and communication practices of many deaf^1^ Tibetans in Lhasa, the capital of the Tibet Autonomous Region (TAR).^2^ At the core of these has been a formalisation of various indigenous signs, gestures and other kinds of signed communication into TibSL – a process that began in 2000 and was led by a group of deaf and hard-of-hearing Tibetans. A signing community has since emerged in relation to this language, with Sonam as one of its actors. This article presents and analyses findings from a short ethnographic study with deaf and hard-of-hearing TibSL signers in 2007 in Lhasa, as well as of TibSL-related language materials, policy and NGO documents collected in 2007 and during a short follow-up visit in 2014.^3^ In Section 1, I will offer glimpses into the brief history of TibSL, its promotion and intermittent teaching at the local deaf school. In Section 2, I present a preliminary assessment of TibSL’s current linguistic vitality and endangerment by discussing nine factors identified by UNESCO and recently adapted for signed languages in the iSLanDS scheme (Zeshan et al. 2011). My findings are then compared to the situation of other minority languages in Tibetan areas of China, and some suggestions made for future research, these together comprising Section 3.

So far, no long-term anthropological or linguistic study on deaf Tibetans in the “Chinese Tibetosphere” (Roche 2014) has been carried out. The main source for this article has been a three-week ethnographic pilot study in 2007 in Lhasa, where I was a long term resident already interested in developing a larger and longer-term future study. I worked mainly with the core group of eight Tibetans who were documenting and developing TibSL and who were active in the Tibet Deaf Association (TDA) at the time. I participated in their team gatherings, deaf club social activities, visited the deaf sewing workshop and attended three weekend TibSL classes at the Lhasa deaf school. In addition to many informal conversations I also had four semi-structured interviews with key TibSL activists as well as conversations with hearing Tibetans who have deaf family members and friends. In addition, I collected official documents and whatever language materials had been produced. I learned a few basic TibSL signs and communicated in a very visual way, including through clear lip movements and gestures. In more in-depth communication and interviews, I relied on the assistance of a hard-of-hearing Tibetan who informally interpreted TibSL to spoken Tibetan (there are no professional TibSL interpreters yet). Although my TibSL was very rudimentary during this first stint of research, I benefited a great deal from the rapport built up with the Tibetan signers due to my own father being deaf and his visit to Lhasa prior to the research period. He had practiced TibSL before his arrival and helped with some of the translations as well. Due to my own growing up with deaf family members, one of them a signer and deaf activist, this made me not only an interesting resource for some deaf Tibetans, but also well aware of the numerous communication and educational issues shared among many deaf people world-wide. I would also say that I am more attuned than most to a visual communication style.

This first period of research was then complemented by a follow-up visit in 2014, when I met with the new leadership of the TDA and the incoming generation of TibSL teachers, as well as the Handicap International (HI) team. They updated me on many issues and shared a range of advocacy materials, photographs, reports and current policy documents and practical information. Furthermore, I was also able to pick up more TibSL during this visit.

## 2 The emergence and promotion of TibSL in Lhasa

According to the governmental census from the year 2000, Lhasa’s central, urban area had approximately 200,000 inhabitants.^4^ From government data and NGO records, it can be surmised that about 1% of the total population of the TAR are deaf or hard of hearing,^5^ which means that there were about 2000 deaf and hard-of hearing persons living in Lhasa in the early 2000s.^6^ Approximately 90%, or 1800, of these can be assumed to be of Tibetan ethnicity. Due to TibSL’s almost exclusive use among deaf Tibetans (and not Han Chinese), this group is taken in this article as the “reference community” for TibSL, further defined and discussed in Section 2. Considering the relative absence of any sizeable urban centres in historic Tibet, this means a much greater number of deaf and hard-of-hearing Tibetans now live in close proximity to each other than probably ever before.

Reports of NGO employees and TibSL activists suggest that when the TibSL project began in 2000, not every deaf or hard-of-hearing person in Lhasa communicated mainly or only in sign. Far from it. Communication choices and possibilities for deaf people of different ages and social groups in Lhasa have been and continue to be just as diverse as in most other places in the world. These comprise a spectrum encompassing exclusive use of sign, communication through speech and lip reading (sometimes assisted by hearing aids), as well as communication in Tibetan or Chinese writing, gestures, and/or a combination of all of the above. In other words communication is deeply multi-modal. The choice of modality often depends mainly on whether one’s communication partner is deaf or hearing and the age of deafening. Comparable to most other places, with the exception of those with an unusually high incidence of hereditary deafness, the vast majority of deaf Tibetan children are born into and live within hearing families. Most deaf people come to learn and work in a largely hearing environment, with members rarely learning a good level of sign language and thus communication often posing a major hurdle in daily life. Furthermore it is relatively rare that people who lose their hearing in older age learn to sign, and this was also the case in Lhasa.

Among those Lhasa Tibetans who did use sign communication, at least some of their time and with other deaf Tibetans, a good degree of diversity of signing practices has been reported from those involved in the early work. Although there are more generally age and gender differences in signing practices, here the differences were perceived to be due mainly to the geographical origins of deaf people who were now living in Lhasa. This situation was seen as a key stimulus for documentation and to a certain extent standardisation of TibSL vocabulary (TDA 2011: 7–8). Many of the signers I met in 2007 and 2014 had themselves moved to Lhasa in search of better work and social opportunities and only there came in contact with other Tibetans who signed. At home and in their native villages or pastoral areas they used relatively restricted sign and other communication, often relying on simple gestures and signs made up within the family in order to communicate daily necessities and affairs with hearing family members or neighbours.^7^ Communication was hence largely built on shared body language of both hearing and deaf Tibetans and therefore relatively limited, again also depending on the age of deafening and other forms of communication used. Only in slightly larger towns or villages, such as Shigatse, Samyé or Tsethang, had there been more developed sign communication among the deaf. To what extent these differed from each other, beyond lexical items merits further linguistic research. It is also likely that these different language practices continue to some extent within the signing population in Lhasa and especially in other parts of Central Tibet, but this does not seem to be seen as a problem. Rather a certain standardisation was perceived as important mainly for the potential use of TibSL in school but not for daily interaction between deaf signers.

**Figure 1:**
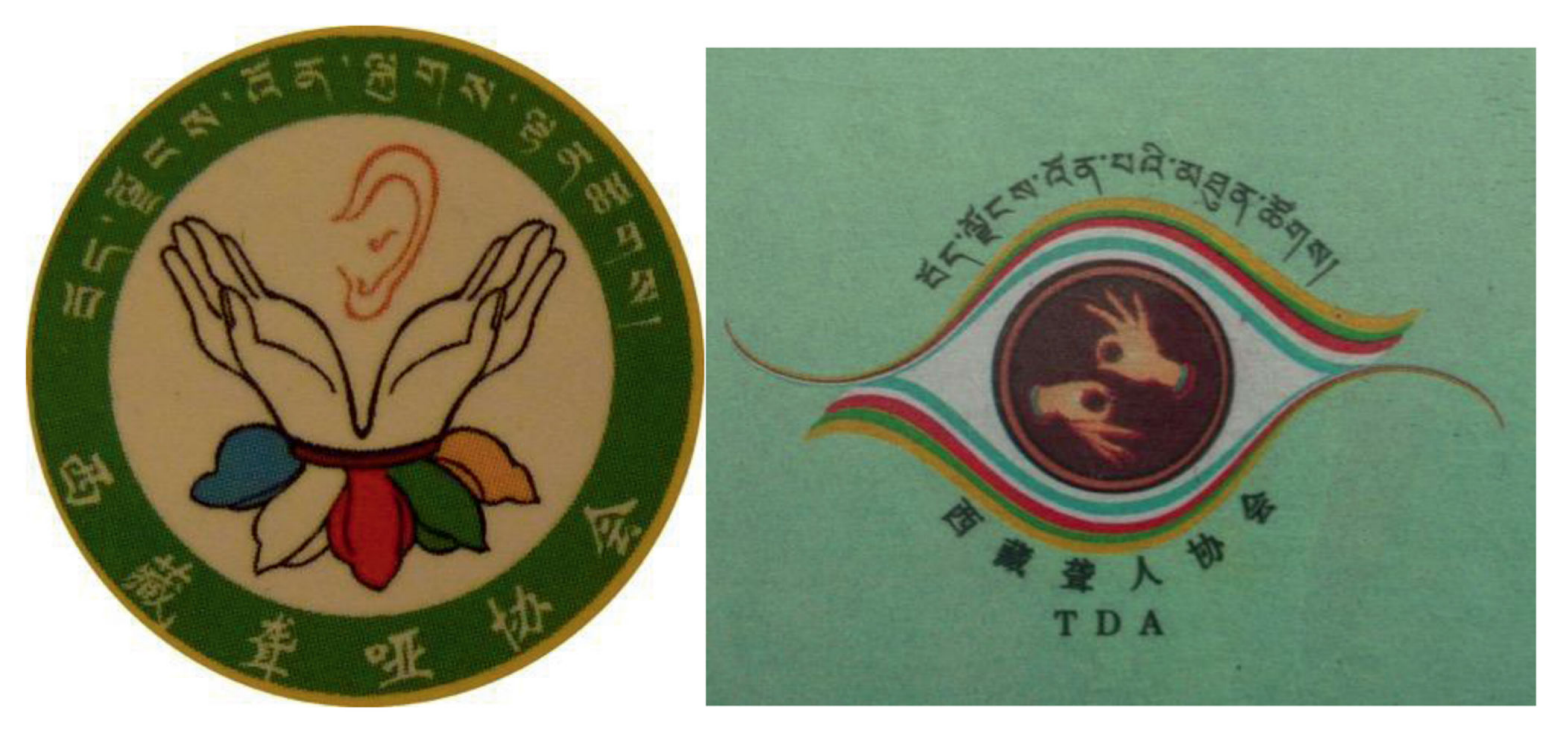
Logos of Tibet Deaf Association from 2004 (left) and 2011 (right).

The overall attempt and mission of the TibSL project in Lhasa was to develop and enhance a native sign language that would have the potential to serve as a lingua franca among Tibetans in Lhasa and among all deaf Tibetans in the TAR (TDA 2011: 7). One that could be used in education and thus build, connect and support a Tibetan deaf community and help its members “participate in the community as any other person” (Sauboin 2005). The main supporter and to some extent initiator of the TibSL project was Handicap International (HI). This international NGO started working in the TAR in 2000, their projects spanning orthopaedics, physiotherapy support, community-based rehabilitation services as well as support for deaf people. In 2001 HI promoted the founding of the TDA (see Figure 1),^8^ a semi-independent association under the helm of the governmental Tibet Disabled People’s Federation (TDPF) and initially part-financed through HI.^9^

The TibSL project was the first and main activity of the TDA. It officially began when TDA founding members were offered rooms on the premises of the Lhasa Community Rehabilitation Services Centre in the east of town and they gained a small but stable salary through the association. At various points the group met with, and was inspired by, foreign visiting consultants and volunteers working with HI. On several occasions this included deaf visiting consultants, who had themselves benefitted from the enormous gains made in deaf education in other countries. These encounters helped to foster more self-confidence in TDA members, who all have had their experiences of societal stigma and discrimination.

The core activists of the TibSL project were initially four Tibetans, who worked at the Centre between 2001 and 2004, their aim being “to formalize the Tibetan sign language (TSL) through the collection of existing signs and the making of a dictionary” (Sauboin 2005). In meetings as well as on visits paid to deaf people in different parts of the TAR, they selected signs from these regions (for instance agricultural and pastoral terms) as well as from among their own geographically-diverse group, subsequently discussing their preferences (cf. Suo and Sun 2008) usually among a group of about 20 deaf Tibetans as part of the Sunday deaf club. Having decided which signs seemed most suitable to the group, they then documented these in video recordings.

**Figure 2:**
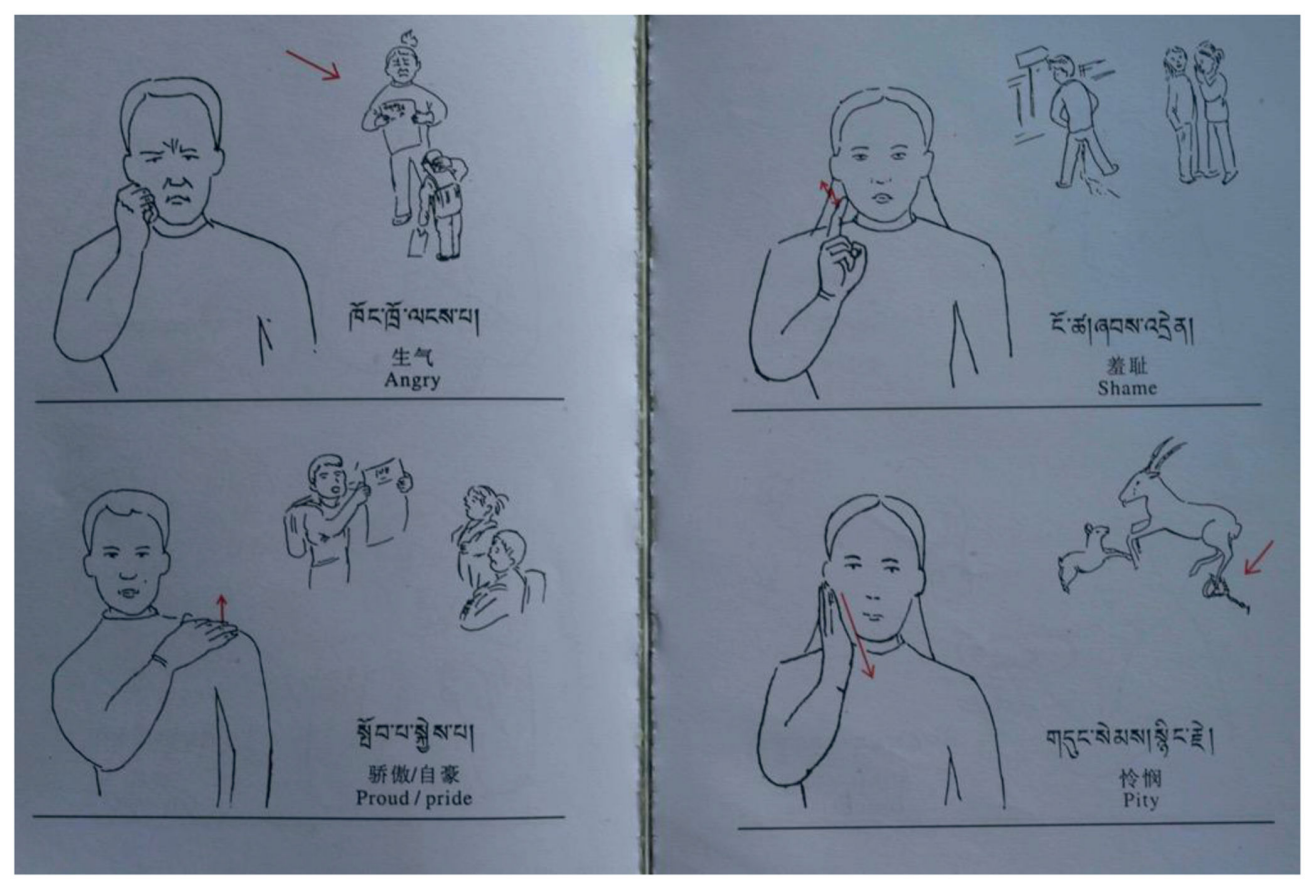
Sample pages of the *Tibetan Sign Textbook* (TDA 2005).

The main outputs of the early years of the TibSL project were two DVD volumes of the *Tibetan Alphabetical Sign Dictionary* (TDA: n.d.), which detail roughly 700 signs structured from *ka* to *A*, following the Tibetan alphabet. Each was furnished with a Tibetan gloss. Several hundred copies were distributed by the TDA for free, including to those attending the TibSL courses taught by the TDA. Owing to the impractical nature of the DVD dictionary (it had for instance no “search” function), work began on textbooks and children’s books. Signs were drawn for these books and the overall number of collected signs was expanded to 827. As a result three volumes of the *Tibetan Sign Language Book* (TDA 2005) were published. In these, signs are grouped together according to domain of life (for example family, fruits, colours, feelings, seasons and weather etc.) and each of the drawings is glossed in Tibetan print letters, as well as translated into Chinese and English (Figure 2).

**Figure 3:**
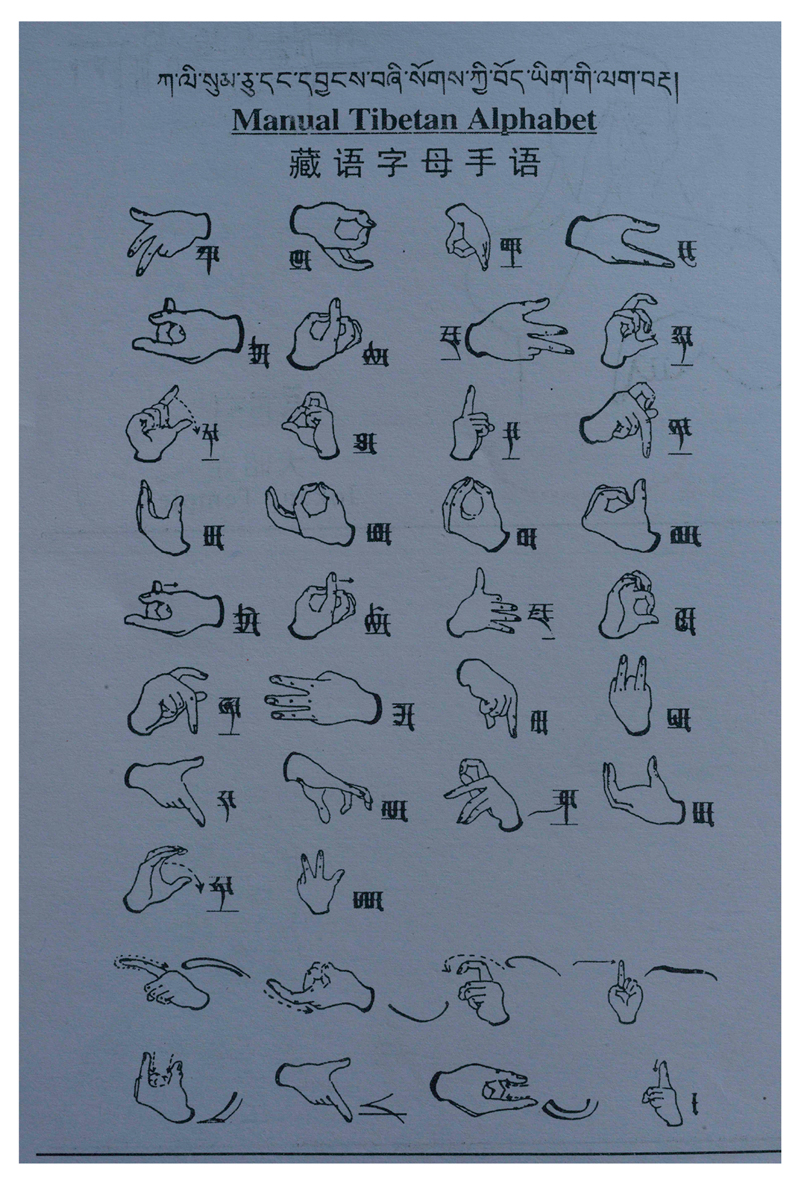
The Tibetan Sign Language finger alphabet (TDA 2005: 100).

These volumes were again given out for free. During the work, the need for a Tibetan finger alphabet had arisen and led to the birth of the TibSL manual alphabet (see Figure 3). It follows and honours the shape and form of the 30 consonants, 4 vowels and the main subscripts of the Tibetan alphabet, as written in *u med* (“headless”, or cursive) script and shows no influence from either IS (International Sign) or American Sign Language (ASL) finger spelling, which both relate to the Roman alphabet used to write many European languages and are therefore of no use. It replaced an earlier finger alphabet developed in 2000/2001, which used two hands for seven out of the thirty consonants, but it was subsequently considered impractical in daily use.

In 2007 I witnessed the teaching of TibSL at the Lhasa Special School^10^ over the course of three consecutive weekend classes. The school, where almost all the pupils were either deaf or hard of hearing, was otherwise a Chinese Sign Language (CSL) and spoken Chinese learning environment, thereby in part following the national policy on deaf education (Johnson 2003; Callaway 2000: 68–72; Lytle et al. 2005/2006). Chinese Sign Language (CSL) basically follows spoken Chinese word order and contains many signs related to Chinese pictograms. Like other primary schools in Lhasa (Bass 1998; Ma 2014), the curriculum also included a few hours of Tibetan language teaching every week, which were here taught by the regular, hearing government teachers, yet focused on oral explanations and pronunciation, and were therefore very hard for the Tibetan children to follow and benefit from. None of the regular teachers signed TibSL, despite some having completed “special education” teacher training. Some teachers reportedly made an effort to use gestural communication that is shared between deaf and hearing Tibetans, a phenomenon some deaf Tibetans refer to as “spontaneous sign” (*rang byung gi lag brda*). On the weekend classes however, three TDA activists who doubled as TibSL teachers came to the school and each taught one of the three different age groups. Through often playful sessions learning TibSL signs and fingerspelling of the Tibetan alphabet, the children expanded their TibSL repertoire yet at the same time learned in a natural and more effective way to read and write Tibetan (see Hofer and Sagli 2017 and Figure 5).

### 2.1 What’s in a name?

**Figure 4:**
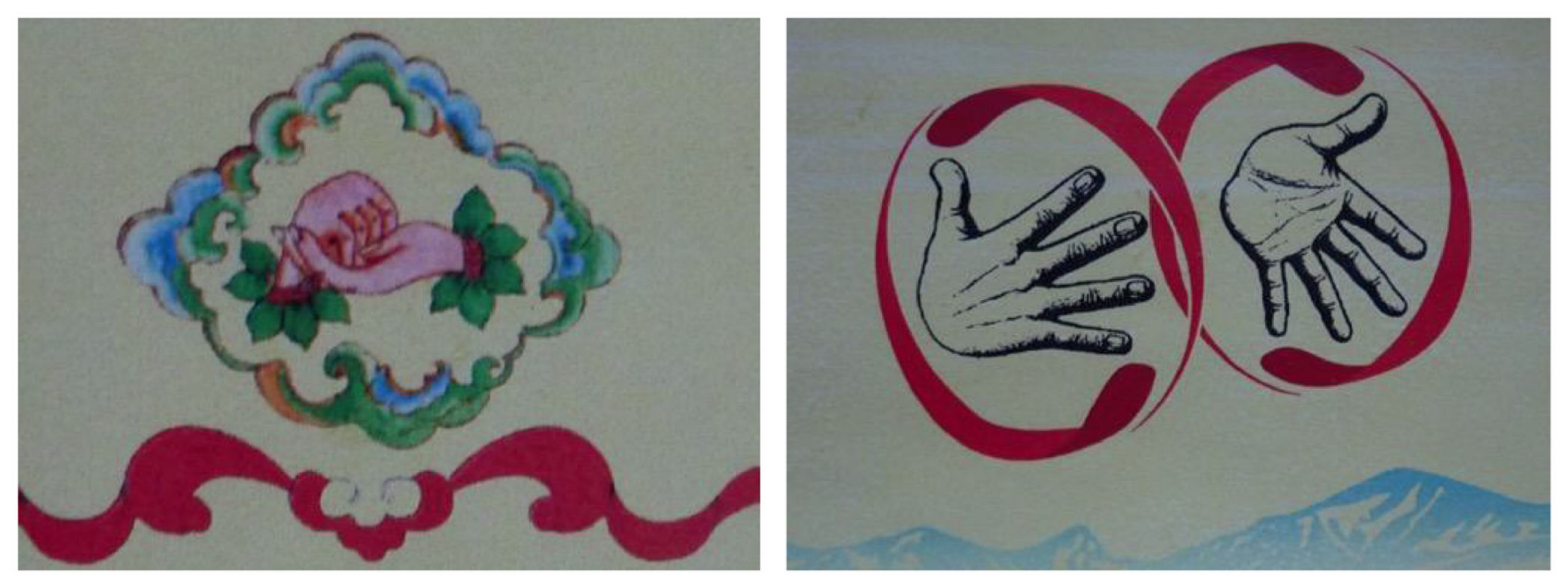
The front and back covers of the *Tibetan Alphabetical Sign Dictionary* showing TibSL for TIBETAN (left) and TibSL & IS for SIGN (right), together constituting the TibSL sign *BÖKYI LAGDA* or ‘TIBETAN SIGN’.

To be sure, the term “Tibetan Sign Language” or TibSL, is an outsider’s term for a language that began to be documented and formalised in Lhasa in 2000. It came into existence in English in 2002 with the publication the first volume of a three-part Tibetan Sign Language Dictionary (HI & TDPF 2002). Here and in the subsequent volumes, as well as the of the three volumes of the *Tibetan Sign Language Book*^11^ in Tibetan this language is referred to as either onkug lagda (‘on lkugs lag brda – in the first volume) or *bökyi lagda* (*bod kyi lag brda* – in the later volumes) literally meaning either ‘deaf and mute hand signs’ or ‘Tibetan hand signs’. These term do not incorporate the Tibetan word for language (*skad*), which in its narrow meaning only refers to spoken languages due to its root being that of ‘voice’ or ‘utterance’. In *bökyi lagda* or TibSL itself, the language is referred to in a combination of TibSL and IS, as shown on the front and back cover of the DVD-based dictionary (Figure 4). TibSL for *bö*, or Tibet – and from now on I will use capital letters to render TibSL signs – is two hands in the action of preparing *pak*, a ball of *tsampa*, the staple food of most Tibetans. IS for SIGN is shown on the back of the DVD andis two hands in a circular motion (Figure 4). The two signs combined make *BÖKYI LAGDA* or TIBETAN SIGN. TibSL signers simply refer to signing with IS for SIGN. In spoken Chinese, TibSL is referred to as the ‘Tibetans’ hand language’ (*zang zu shou yu*).

Whatever terms we find used in the TibSL materials, or in use by Tibetan signers themselves, the majority of even highly-educated hearing Tibetans with whom I spoke were unaware of a sophisticated sign language currently in use among deaf Tibetans in Lhasa, even if some themselves have deaf relatives. If the communication of the deaf was named at all, it tended to be referred as *lkugs brda* ‘dumb or mute’s signs’. While sounding obviously derogatory in English, the Tibetan term can also refers to non-verbal communication, including between hearing Tibetans.^12^ Many Tibetans still use the terms *lkugs pa* or *lkugs ma* ‘dumb, mute, dull’ for the deaf and it was, until 2011, found even in the Tibetan name of the TDA (as part of the term ‘deaf and mute’, ’*on lkugs*, see Figure 1). However, the use of the term *lkugs pa* is slowly falling out of use among educated Lhasa Tibetans as deaf people feel offended by this term. Younger (hearing) Tibetans who knew about sign languages would often use the Chinese term *shou yu* – or ‘hand language’ – adding they did not have a proper term for it in Tibetan.

Terminology aside, and from an outsider’s and a linguistic point of view, should TibSL be conceived of as a language in its own right? What about other sign languages on the Tibetan plateau and what might their relations be with each other and to TibSL?

To date there has been no in-depth linguistic or socio-linguistic study of TibSL (or any other signing practice among Tibetans on the Tibetan Plateau). The question of what the key features of TibSL, or any other form of signing by Tibetans, are is currently open and requires considerable further research. Yet based on my initial findings presented here, the analysis of the language materials, and most importantly the TibSL signers’ own perceptions and my observation of their use of TibSL in daily communication, there are indications that it is a language in its own right and an application for an ISO-639-3 number for TibSL is currently under way.^13^ While there may be considerable overlap with other native Tibetan signing practices – potentially but less likely also in Exile – the language clearly differs from Chinese Sign Language, or CSL.

Literature on the history of CSL, China’s internal sign language diversity and current issues relating to the use of both among the deaf in China is scarce. An important question arising from this is to what extent CSL really is the language of the deaf in China. Many Chinese scholars take it to be so (cf. Yang and Fisher 2002; Wei and Dinqian 2014), while outside observers tend to understand it as an artificially created code propagated by the state, but one often poorly understood and not widely used by deaf Chinese (cf. Callaway 2000).^14^ Callaway holds that CSL resulted mainly from concerted standardisation efforts begun in the 1950s and again during the 1980s, and that these were led mostly by hearing Chinese (Callaway 2000: 82–88). Be that as it may, CSL is represented in national standard CSL dictionaries, taught in deaf teacher training institutes in inland China, and it is used in Sign Language Interpretation (SLI) in over 200 Chinese TV channels (Xiao et al. 2015). In *Ethnologue*, CSL is also listed as one of the languages of China. Despite its history of (at least) in part, top-down standardisation and the ongoing problems that CSL poses for many deaf and hard-of-hearing, China’s official publications refer to CSL as “deaf people’s sign language” (Callaway 2000: 84). Yet, no natural sign language of deaf Han Chinese, i. e. substantially divergent from CSL, has so far been officially recognised. The number of deaf Tibetans using almost exclusively CSL is growing fast due to its use at the Special Schools.

In May 2004 Tibetan sign was recognised by the Chinese government as the first “minority sign language” in China. The state news agency, Xinhua, reported that this “is the first sign language system designed for deaf-mutes of a minority ethnicity in China”, and that “this dactylology system differs from the one being used nationwide as the latter is basically a kind of Chinese character conversion whereas many Tibetans can neither read nor write the Chinese characters” (Xinhua 2004). The language is described here as having been compiled by four members of a Tibetan deaf club based on their collection of local signs and that they were now in the process of popularising the language “among local Tibetan deaf farmers and herdsmen” (Xinhua 2004). The precise status of the official recognition of Tibetan sign, the wording used for the language and its practical implications are not yet clear but what documents I could find are discussed below. According to the TibSL activists, recognition has yet to yield more practical consequences for TibSL and deaf Tibetans, for instance regarding education in TibSL at the Lhasa Special School and others that have since been established in the TAR.

### 2.2 TibSL Documentation, formalisation or standardisation?

In contrast to the mostly hearing agents involved in the top-down CSL standardisation project, the TibSL activists working on the formalisation of TibSL were all deaf themselves, used the language in daily interactions and wanted to promote it. From the situation of CSL as well as from international NGO and visiting sign experts and deaf educators, they nevertheless learned about the powerful impact that language documentation and materials can have on the users of a language, its official status and support.^15^

Questions remain regarding TibSL, relating to the details and aims of the recent documentation and formalisation efforts, where and by who TibSL is actually used in Lhasa and beyond, and the extent of its internal diversity, be that due to signers’ variable geographic origins and/or other social distinctions. A tension commonly recognised by sign linguists is that even the best efforts in creating sign dictionaries and language materials – i. e. efforts to standardise the language – do not necessarily also mean that the language is therefore more widely used and spread, although these efforts are generally effective in raising the official recognition and visibility of the language.^16^ It is therefore important to get a better understanding of how many people in fact use TibSL and how their number is changing over time. To tackle these and related questions and issues, a thorough linguistic and socio-linguistic study of various forms of Tibetan sign and TibSL is warranted, alongside more and longer-term ethnographic research.^17^

From my preliminary study, the most fluent signers of TibSL are the TibSL and TDA activists themselves, as well as those who have been attending signing classes or are otherwise involved in the TDA’s activities in Lhasa. The language was used among deaf people in several “deaf spaces” that existed in Lhasa in 2007, including the TDA-initiated sewing workshop (established in 2004), a deaf social club offering a chance for deaf Tibetans to come together, exchange information and gain hands-on TibSL practice, as well a deaf space created during the weekly TibSL classes at the Tibet Disabled Persons’ Vocational Training Centre (TDPVTC). To my knowledge, in 2007 no TibSL classes or formal deaf spaces existed outside of Lhasa.

We now turn to a more detailed discussion of the current linguistic vitality of TibSL in Lhasa and in Tibet more broadly, information gathered from a short follow-up visit I made to Lhasa in early December 2014, which added significantly to my 2007 findings.

## 3 Is TibSL an endangered language? The UNESCO model and linguistic vitality and endangerment of signed languages

Realising the speed and extent of loss languages across the globe over the past three decades, a large number of linguists and anthropologists have turned towards language documentation. In the process they also often engage in language revitalisation to preserve linguistic and cultural diversity. Different models to assess the degree of language vitality and endangerment have been proposed and used (Dwyer 2012). Here I follow UNESCO’s model, which is based on nine dynamic factors, outlined in Table 1, and uses grades between 0 and 5, with the higher grade indicating conditions conducive to long-term survival of the language, while 0 evidences the worst possible condition for a language, that is, its extinction (Brenzinger et al. 2003).

The findings from such an assessment have in many cases led to the inclusion of endangered languages in the *UNESCO World Atlas of Languages in Danger* (for short *The UNESCO Atlas*, Moseley 2010). Such listings and assessment have at times had real-world impacts, on state-recognition as well as in relation to community attitudes and language practices, fostering for example more documentation, inclusion in education and other activities promoting the language.

So far no sign language has been included among the 2,400 languages currently listed as endangered in the print and online versions of *The UNESCO Atlas*. On the other hand, the 18th edition of *Ethnologue*, a key linguistic database, includes over 130 sign languages, some of them also small and endangered (Lewis et al. 2015a). Safar and Webster find that compared to many of the world’s spoken languages, “sign languages are in similar, possibly even more precarious situations” (2014: 1). *Ethnologue* has recently adapted its own vitality ranking, so that it can also assess the vitality of signed languages (Bickford et al. 2013). A research project to document, map and assess endangered sign languages world-wide, following the UNESCO model, is currently led and coordinated by the sign linguist Ulrike Zeshan and her colleagues based at the International Institute for Sign Languages and Deaf Studies (iSLanDS), University of Central Lancashire in the UK. Their map currently identifies 15 endangered sign languages, while the team researches many others, with the results envisioned to enter eventually the standard editions of *The UNESCO Atlas*. For this project the team has adapted the UNESCO model to make it more suitable for assessing signed rather than spoken languages, both in the terminology and content of the questionnaire. I have found this a very useful adaptation and offer a comparative table of UNESCO and iSLanDS terminology for the nine-factor classification below (see Table 1). In the coming discussion, while following the UNESCO model in broad terms, with regard to the specific ratings I rely on the iSLanDS adaptation as it presents a much more accurate picture of signed languages and was developed by specialist sign linguists. I will refer to that questionnaire as the *Adapted Survey* (Zeshan et al. 2011).^18^

### 3.1 Linguistic vitality of Tibetan Sign Language

#### 3.1.1 Intergenerational language transmission

Intergenerational Language Transmission is UNESCO’s first factor and one of the most significant elements to assess whether a language is learned and used by community’s elders and children alike. It serves as an indicator of a language being passed on through generations in the home, the primary place of spoken language acquisition. When it comes to sign languages the situation is markedly different. Unless there is hereditary deafness in a family or a village (Kusters 2010), most deaf children do not learn a more developed sign language from their own parents or grandparents in the home. Instead they do so outside the home, usually from peers and/or deaf educators in schools and deaf associations, or even develop their own sign language – which in the case of a newly founded deaf school in western Nicaragua led eventually to the development of the national Nicaraguan Sign Language or ISN (Senghas and Coppola 2001). Bickford et al. (2013) suggest that the key to understanding this factor, therefore, is not necessarily where and from whom a sign language is learned but whether children are learning sign language.

**Figure 5:**
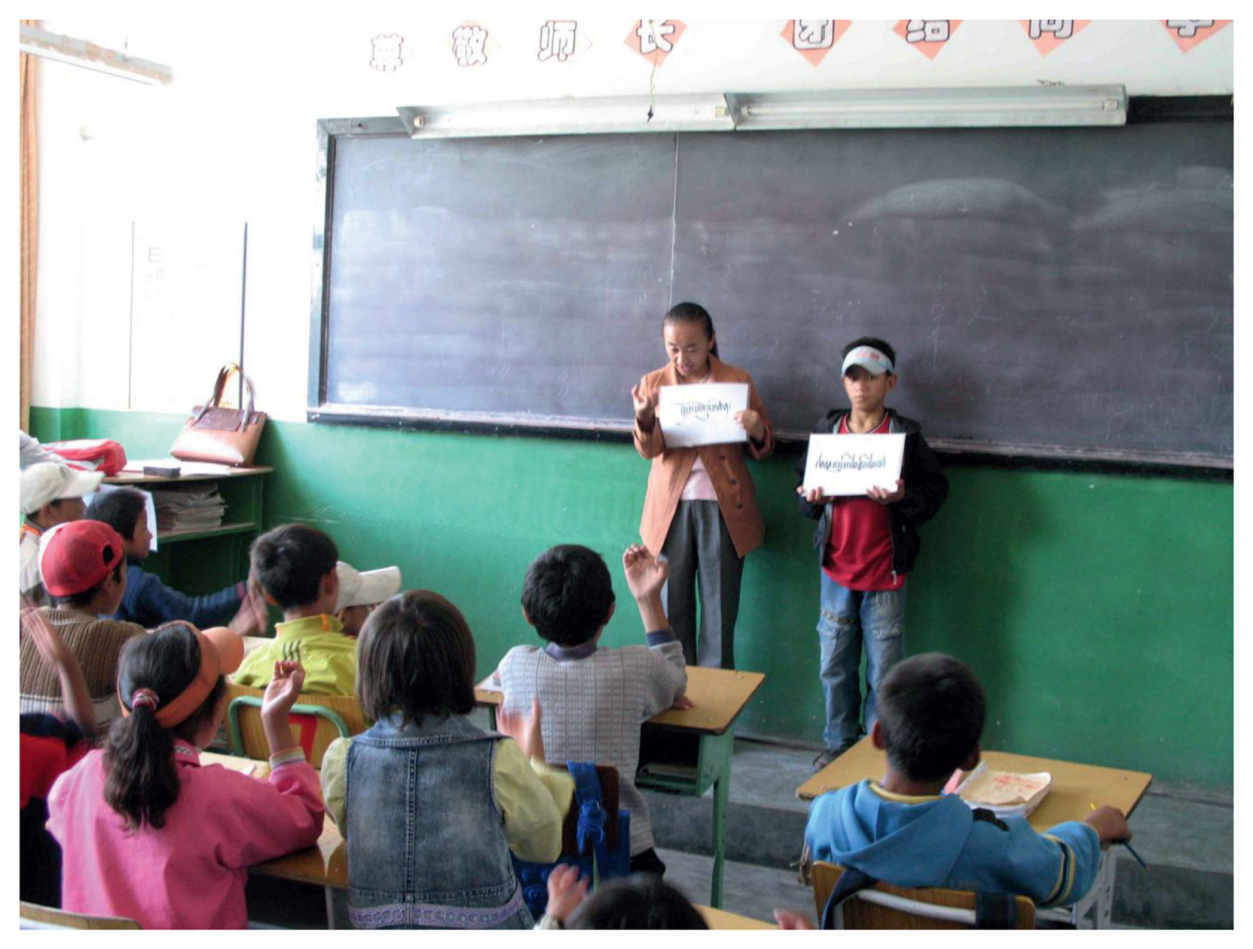
Pupils learning new TibSL signs and finger spelling in an extra-curricular class at the Lhasa Special School, 2007. Photo: Theresia Hofer.

From my observations of TibSL users there are more among the age group of 25 to 40 than above or below. This would be quite natural given the recent emergence of this language and that most of those deafened in old age do not tend to learn sign. The younger generation faces challenges as well. Despite the earlier teaching of TibSL during the weekend classes at the local deaf school (Figure 5), the 2008 Education Bureau’s official recognition of TibSL and its policy statement to make TibSL teaching compulsory Tibetan special schools, as well as important statements made in support of Tibetan Braille and Tibetan sign language by the CPC TAR Party Committee (2010), since 2010 children in the Lhasa Special School have no longer been exposed to TibSL (with exception of some irregular informal visits by TDA members).^19^ The ending of the regular and officially endorsed TibSL weekend classes by the deaf TDA teachers was due partially to an internal conflict in 2009, but also because they simple never really felt welcome by the leadership and teachers of the Lhasa Special School. After 2008 there were also more stringent governmental attitudes and policies regarding Tibetans’ involvement with foreign NGOs and restrictions on a variety of other activities. As a result, and related in part to the changes in teaching TibSL at the Lhasa Special School, the youngest members of the TibSL reference community in Lhasa are currently not learning or fluent in TibSL. This tend to be especially those under the age of 25.

iSLanDS’ *Adapted Survey* qualifies the first UNESCO factor to Generational/Age Group Use, which itself is split into questions on either long-standing sign languages or emerging sign languages. Following the latter, the score I could assign TibSL in this category is 4, or “unsafe/vulnerable”, summarised as follows: “A substantial subsection of age groups from the oldest signer ‘downwards’ uses the sign language competently, but the language is starting to be lost from some age groups e. g. the youngest ones” (Zeshan et al. 2011: 9).^20^

#### 3.1.2 Absolute number of speakers

This factor quantifies the vulnerability of a language to outside forces (such as disease, warfare or natural disaster) based on the community size. iSLanDS adapts this factor to: Number of Sign Language Users. Of the estimated 1,800 deaf Tibetans living in Lhasa, the TDA’s director suggested to me in 2014 that about 300 of them use TibSL in their interactions with deaf friends and work colleagues and some with hearing relatives. Given that the TDA then had 190 active members, who are regularly in contact with other deaf Tibetans, 300 might be a slight overestimate – perhaps an expression of the director’s hope for the language. However, I have no solid data to disprove her. My research stay was too short to assess the number of TibSL users in Lhasa. Although I hope this can be done in the future and by using methods that have been developed elsewhere (e. g. Nonaka 2009), it will likely continue to be difficult in the politically sensitive environment of Lhasa. For the purpose of the current article, I will use the TDA president’s estimate of 300 TibSL signers in Lhasa, and hope to be able to offer more detailed data in the future. This way to proceed seems permissible, as the total number of language users is not a particularly clear indicator of vitality or endangerment: many large languages have disappeared, while some small languages can and do persist.

The main outside “threat” to the numbers of signers, especially in Europe and the US, has been improved medical care and the spread of the Cochlear Implant (CI), a high-tech surgical intervention that can enable some hearing in both infant and adult populations. The CI has often resulted in a smaller perceived need for sign communication, at least by parents, doctors and government. Neither improved medical care nor audiological technology are, however, currently especially strong factors reducing or limiting the signing population of Lhasa. Medical care in the TAR, in rural areas particularly, has not actually improved much since the late 1990s, with ototoxic antibiotics tending to be wrongly prescribed and overused by minimally-educated health workers and pharmacists often leading to deafness (Hofer 2011; cf. Callaway 2000). Even in Lhasa, for those outside of government work units, medical care is expensive and sometimes unaffordable. Specialised ear-doctors and audiologists are almost non-existent. The overall number of the deaf has therefore not substantially reduced, I would suggest. Rather, the Lhasa-based total deaf population has likely increased over the duration of the TibSL project, due to urbanisation and migration – almost half of TDA members coming from outside of Lhasa (HI 2011: 15). CIs or the use of simple hearing aids have also not so-far reduced the Tibetan signing population, although, as in the rest of the PRC, both will probably become more popular in the near- and mid-term future, given the pervasive medicalisation of deafness and the CDPF’s active promotion of medical and technical solutions (Callaway 2000; Kohrman 2005). Out of twenty-one TDA members interviewed for an HI survey in 2011, only three used hearing aids. In addition, I have heard of others not being able to find the right batteries in town after having been given expensive state-of-the-art hearing aids and almost immediately having to abandon their use.

#### 3.1.3 Relative proportion of signers

The next iSLanDS criteria is the relative proportion of signers within the reference community. Here a definition of the reference community of signers is first warranted. In sign languages this has to be carried out on a case-by-case basis, as conventional criteria for spoken language reference communities – such as ethnicity, heritage, culture or geography – do not necessarily determine the group who in this case would be “expected to use sign language” (Zeshan et al. 2011: 5). To determine the overall size of the reference community of TibSL, my starting point was the total Tibetan deaf population of Lhasa, which, as earlier stated, can be estimated to be about 1,800. To deaf signers, according to Zeshan et al., should normally be added many other signers. In many European settings these would include hearing children, siblings, spouses and other relatives of deaf adults, as well as other groups of hearing people in regular contact with the deaf, such as neighbours, professional sign language interpreters, co-workers etc. For the case of TibSL, I would like to propose that the total reference community remains limited to the total number of deaf Tibetans in Lhasa, i. e. approximately 1,800. The reasons for this are manifold, including: TibSL being a recently emerging language; that it arose in a vibrant urban centre rather than an isolated village community with high hereditary deafness (cf. Zeshan and de Vos 2012; Nonaka 2009); that the stigma of deafness persists and few parents of deaf children learn to sign; that the local deaf school has not trained its own teachers in TibSL; and that there are no professional sign interpreters in Lhasa. This means that the 300 TibSL signers estimated by the TDA president in 2014, make up about 10–15 % of the Lhasa-based reference community. Hence I admitted a score of 1, or “Very few use the sign language (<30 %)”.^21^

#### 3.1.4 Domains of language use

This category indicates the range of topics and areas of life where the language in question is employed, which directly affects whether or not it has a chance to be used in the next generation or not. Working with iSLanDS’ questionnaire, I suggest that TibSL currently enjoys “multi-lingual parity”, or a score of 4, qualified in the following way: “two or more (signed and/or spoken) languages are used in most social domains and for most functions; the use of the *target sign language* [i. e. in this case TibSL] is usually rarely in the official domains (e. g. government, business, administration, formal education etc.) but is very present in the community’s public domains (e. g. deaf school dormitories, community gatherings etc.) and in informal domains (e. g. in families)” (Zeshan et al. 2011: 10). The reason for my score is mainly that TibSL is the language used in all TDA-organised gatherings, such as the Friday TibSL classes, the Sunday deaf club, as well as informal domains, such as picnics with friends.

#### 3.1.5 New domains and media

Here only one notable development has taken place so far, namely the sharing of TibSL videos via WeChat, a Chinese social media outlet similar to Facebook (which is not available in China). Given this situation, and so far no TibSL interpretation on TV, I would score this as 2, i. e. “the sign language is rarely used in new domains”, an assessment between “used sometimes” (3) and “never used” (1).

#### 3.1.6 Materials for language education and literacy

Regarding the sixth UNESCO factor, we need to remember that almost no sign language has widespread written forms even when they have strong support from governments and education systems (cf. Bickford et al. 2013). iSLanDS therefore refers to Materials for Language Spread and Education.

**Figure 6:**
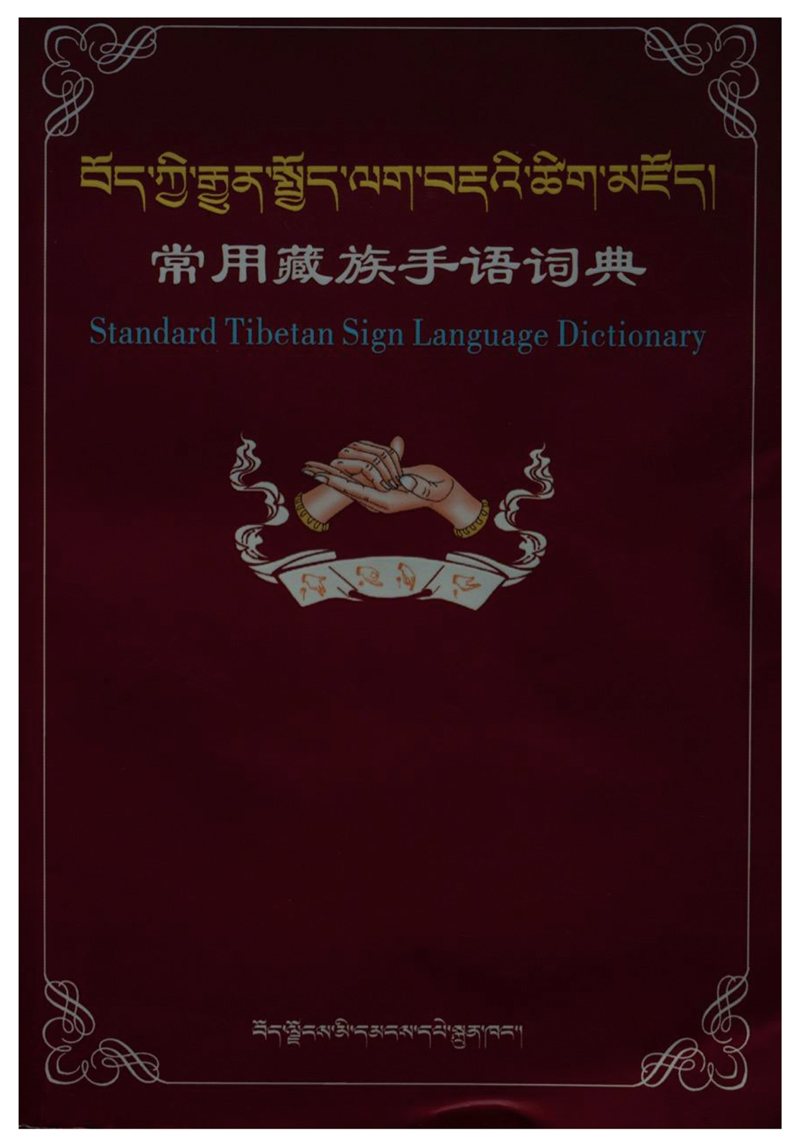
Front cover of the *Standard Tibetan Sign Dictionary* (TDA 2011).

Thanks to the financial and professional support received by the TDA and the TibSL project part-supported by HI, the TDPF and through funding from different development collaborations, a solid range of materials have been produced on TibSL. In addition to the ones mentioned in the introduction, in 2011 the so-far most comprehensive *Standard Tibetan Sign Language Dictionary* was published (Figure 6). It includes most of the 827 signs from the earlier volumes and an additional 610, bringing the total to 1,437 signs (TDA 2011). Published by the governmental “TAR People’s Publishing House”, it has received official acceptance and in the process had engaged several different stakeholders and national and international sign experts. Local government offices (in addition to TDPF), such as the TAR Education Bureau, the TAR Language Committee and the Lhasa Special School were involved, demonstrating relatively high-profile and official support. The dictionary is again organised according to domains of life, yet this time features an expanded introduction as well as an appendix explaining – in Tibetan, Chinese and English – some key grammar rules of signed languages in general, and of TibSL in particular. It is one step closer to being usable also as a TibSL textbook, rather than just a dictionary. The quality of the sign representation has also been improved, featuring real photographs that have been professionally edited, using arrows for movement. Running to an impressive 654 pages, it is available for only 35 RMB (just under 6 USD) in Lhasa bookshops and was in 2014 easily available and affordable.

The strong language materials that have been produced on TibSL so far, in such a politically sensitive context and by a group with relatively low levels of education and training, are remarkable and should warrant a high score. However, this has to be weighed up against the fact that there have been no classes in TibSL at the Lhasa Special School in the last few years. That said, several regional and national documents have, at the same time, started to encourage the use of TibSL in the Tibetan Special Schools. There was in 2014 hope that the lack of TibSL at the Lhasa was just a temporary situation and will eventually make way for the actual implementation of the recent national policies mentioned above. With the good quality video- and book-based TibSL language materials in mind and despite the current, almost total, absence of TibSL from schools, I have assigned a score of 3, qualified in the *Adapted Survey* as: “Some video materials exist and children may be exposed to the language at school, but sign language is not promoted through mass media” (Zeshan et al. 2011: 11).

#### 3.1.7 Governmental and institutional language attitudes and policies

In this area of assessment, which also included Official Status and Use, I cannot be overly optimistic at present. There appear to be some positive official policies, but these are not implemented locally. While TibSL was recognised as the first “minority sign language” of China in 2004 (Xinhua 2004; Suo and Sun 2008), it remains unclear at what level this took place. That it was “real” can be seen in the 2008 Education Bureau’s regulations and stipulations on TibSL use in Special Education in the TAR. Moreover, it featured in the 2010 CPC TAR Party Committee statements, which favour support for Tibetan sign language in education (p. 10), sign interpretation in prefectural and city level TV news (p. 17), and stipulate that Disabled People’s Federations should provide free Braille translation and sign language interpretation in legal disputes (p. 22). However, the current lack of support for the language at the Lhasa Special School and the recently unreliable and unpredictable attitude of the TDPF towards the TDA’s TibSL courses, mean that in iSLanDS’ terminology I would rate TibSL status as 4, or one of “differentiated support”: “The natural sign language is protected primarily as the language of the private domain. It may be in competition with an artificial signed code” (Zeshan et al. 2011: 11). The iSLanDS assessment further adds to this factor a subsection on the Use of the Target Sign Language in Deaf Education, which for TibSL should be described as a hybrid situation that doesn’t fit either of iSLanDS’ scores of 2 or 3. Despite the above-mentioned promising policy documents, the policies are not implemented in deaf schools, and the target sign language is not used at the school, where attitudes are largely negative towards it. In Table 1 I assigned grade 2, mainly based on the current lack of TibSL at the school.

#### 3.1.8 Reference community members’ attitudes towards their own sign language

This iSLanDS’ criteria and community members’ attitudes are generally seen by linguists as one of the most influential factors determining the fate of any language. Given the long-term and enthusiastic involvement of many deaf Tibetans in the TibSL project, as well as their subjective experience of improved communication through TibSL, the score would be very good for the TibSL users within the reference community. Yet here the whole reference community needs to be taken into account and, as we have seen, active TibSL users only make up 10 to 15 % of that community and even among them, especially among the youngest, we find considerable use of CSL. The score assigned therefore can only be that of 2, or “Some members support language maintenance; many are indifferent or may even support language shift” (Zeshan et al. 2011: 12). More substantial information on the attitudes of the remaining 85 to 90 % of the reference community in Lhasa would be highly valuable to be clearer on their perspectives. I think their non-use of TibSL (or any other sign system) is not necessarily their own choice, but rather connected to a lack of knowledge about TibSL and/or being surrounded by a more negative than positive attitude towards sign language and/or deaf people.

#### 3.1.9 Type and quality of documentation

The last UNESCO factor evaluates the overall quantity and quality of written texts, including transcribed, translated and annotated audio-visual recordings of natural speech. The scores here are direct indicators for the urgency of language documentation. In iSLanDS’ *Adapted Survey* I admitted a score of 2. This was a difficult decision, ultimately determined by the absence of an adequate grammar despite there being video recordings, albeit not many of natural TibSL interactions (rather of more formalised teaching and presentations made in the context of the TibSL project and language material creation). Scoring 2 here denotes that language documentation is “fragmentary”, i. e. “there are some grammatical sketches, wordlists, and texts useful for limited linguistic research but with inadequate coverage. Video recording may exist in varying quality, with or without any annotation” (Zeshan et al. 2011: 13).^22^ To improve language documentation for TibSL will require, but is not limited to, the creation of a TibSL grammar, extensive recording of informal conversations and gatherings, as well as an increased use of videos of daily communication and life by TibSL users. And, that all of these materials are made available to linguists.

**Figure 7:**
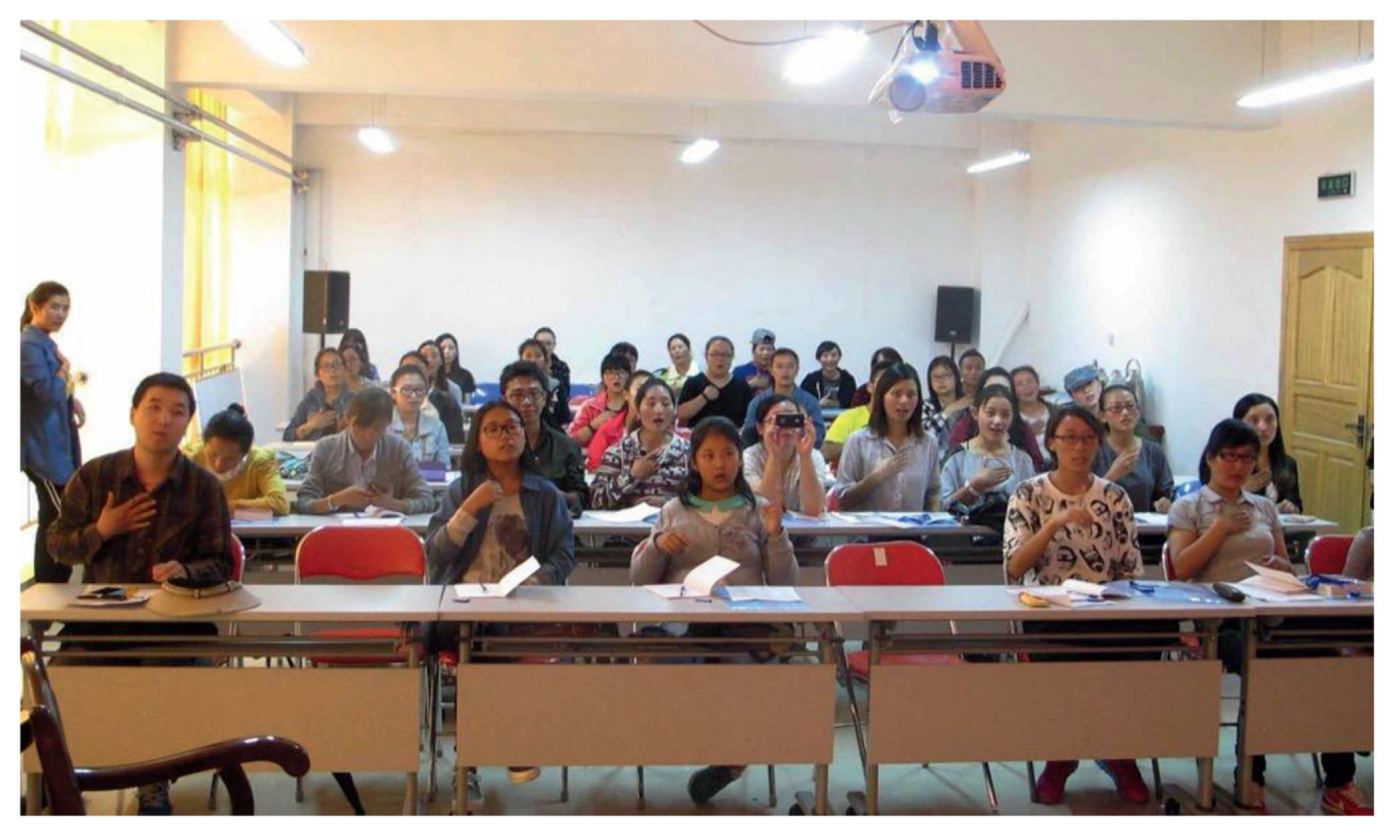
Students of a TibSL course held in Lhasa, summer 2014. Photo: Courtesy of TDA.

iSLanDS adds a further criteria, namely the Status of Language Programmes, knowing that many signers depend on these more than when learning spoken languages. This additional element refers to programmes aimed at promoting the use and maintenance of the language, ranging from summer schools to sign language competitions and regular language classes. I would give this a score of 2, meaning basic, and that “a programme is running involving <5 % of the community, irregularly and with few or no outcomes” (Zeshan et al. 2011: 13). The main reason for this score is that only 10–15 people regularly attend the Friday afternoon TibSL courses, moreover other more intensive (summer) courses were recently blocked or delayed by the TDPF (although they eventually allowed a ten-day training course to occur, see Figure 7).

### 3.2 Discussion

In five out of the nine UNESCO factors discussed and assessed above, TibSL scores between 1 and 3. This is rather low on the scale, indicating that the language is either “definitely, severely or critically endangered”. Slightly more positive scores only concern Generational/Age Group Use, Domains of Language Use and Official Policies, which are rated 4, or “vulnerable/unsafe”.

The overall score within eight of the nine UNESCO factors as adjusted by iSLanDS and minus Absolute Numbers of Speakers, comes to 2.75, i. e. between “severely endangered” (2) and “definitely endangered” (3). It comes to 2.6, when taking into account the additional two iSLanDS sub-factors (again minus the Absolute Numbers of Signers, bringing the total to ten factors, see Table 1). These scores and their discussion here are based on preliminary research and a more definitive assessment will require further research and documentation of TibSL and its use in the future. Yet, so far, the current article presents useful information on the currently limited vitality of TibSL in Lhasa, as well as offering a possibility to account for changes over time.

**Figure 8:**
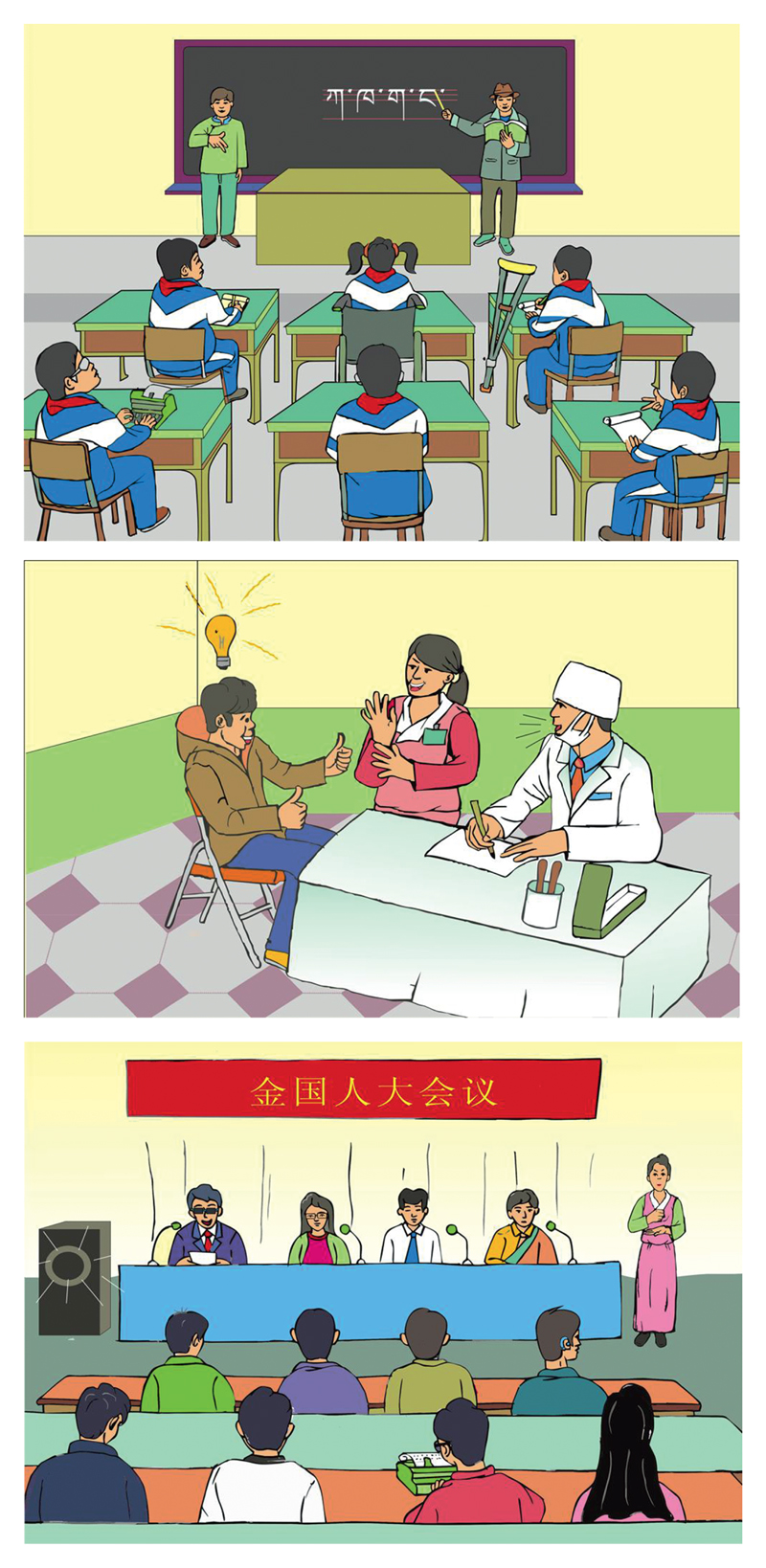
Artworks by a Tibetan artist to advocate the use of sign language in school, medical settings, and politics. Courtesy and copyright: Chogoen and HI.

There are several important developments and agents that are likely to continue to exert significant influence on the prospects of TibSL, but that are not adequately assessed within the framework of the UNESCO and iSLanDS models. For example, one issue here is the different impact of urbanisation and voluntary migration on signing and speech communities. In the case of TibSL, urbanisation and migration have been a positive force for the emergence and expansion of the signing community, while urbanisation is often described as a death sentence to spoken minority languages (although there are some exceptions).^23^ Another factor that is not really captured in the UNESCO model is the influence from international legal frameworks and discourses. It is clear that China’s signing of the United Nations Convention on the Rights of People with Disability (UNCRPD) has led to new legislation and policies at several levels, and even in the TAR as is evident in some of the government documents mentioned above. But there also have been important influences from the international Deaf community on deaf Tibetans in Lhasa, both through the advocacy of HI but also sometimes personal encounters and training opportunities abroad. The TDA’s current leadership also participates in national Chinese Deaf gatherings and there were introduced to international and national commitments in the arena of sign language use, deaf education and sign interpretation (Figure 8). Here they are also exposed to and learn Chinese signs. In other words, it would be fruitful to complement the UNESCO model and its iSLanDS adaptation for sign with more nuanced discussion and evaluation of the complex interaction of, what Grinevald (2014) has usefully distinguished as the academic, local, national, and international spheres of language ideologies.

## 4 Conclusion

Deaf Tibetans in Lhasa find themselves in a complex and dynamic linguistic and socio-political situation. Roche has rightly observed that minority language speakers in the Chinese Tibetosphere tend to be “minorities twice over”, that is, they are classified as part of a *minzu* (an ethnic minority, or ‘minority nationality’) within the Han-dominated PRC state, as well as a linguistic minority within the Tibetan *minzu* (2014: 21). I would suggest that some of the TibSL signers are “minorities thrice over”. In addition to being members of the two minorities mentioned by Roche, they also belong to a minority sign community within a vast state-sponsored CSL and Chinese domains which exert dominance in whatever little exists of deaf activism and sign language-based deaf education within the PRC, even in minority areas that otherwise have special minority (spoken) language policies. Bickford et al. (2013) suggest that sign languages tend to be more resistant to encroachment from dominant spoken languages, but are fragile in the face of dominant sign languages. This is also supported by several other studies (e. g. Zeshan and de Vos 2012; Zeshan and Dikuyuva 2013). In Lhasa and the TAR, especially in the realm of deaf education at the Special Schools, CSL currently has a dominant position and exerts pressure on the growth and use of TibSL in the Tibetan deaf graduates from the Lhasa school sign more CSL than TibSL and have in recent years apparently connected little with the wider TibSL community after leaving school. A further increase of CSL use among Tibetans should be expected. CSL’s role in deaf education is strengthened in the PRC, mainly through the national deaf teacher training courses and selected deaf colleges where CSL is used in of instruction (cf. Lytle et al. 2005/2006; Lewis et al. 2015b: 21). Some China-wide deaf organisations are also trying to reclaim this language as “their own”, using it at national deaf gatherings and conferences in their campaigns for deaf people’s rights. According to *Ethnologue*’s assessment, CSL is “developing”, scoring 5 in their eight-fold Expanded Graded Intergenerational Disruption Scale (EGIDS) ranking of linguistic vitality (Lewis et al. 2015b: 21). Unsurprisingly, perhaps, there are also imports from CSL into TibSL within daily use, for instance signs for countries and technical innovations. Such linguistic borrowings are, it should be noted, also common among Tibetan speakers in Lhasa.^24^ Yet, the import of CSL signs into TibSL seems to follow very different patters from spoken Lhasa Tibetan.

At the same time, it is also possible that TibSL – if its user group continues to expand in Lhasa and beyond – exerts pressure on localised, regional, or even village sign languages that may exist across the Central Tibetan region. If so, we would not know, as linguists and anthropologists are not currently aware of these. This is commonly the case where urban-based deaf communities are struggling to formalise and legitimate their regional or national sign language and are therefore keen to keep diversity and competing interests at bay. In this case it would reflect the situation of other minority language speakers across the Tibetan Plateau, as they are also exposed to strong rhetoric by powerful promoters (Lamas, teachers and others) of the ‘pure father tongue’ or ‘pure Tibetan’ (*pha skad gtsang ma* or *bod skad gtsang ma*) movement (Robin 2014). Many Tibetans speaking small minority languages or dialects, hence find themselves enjoined to switch from their own mother tongue to major Tibetan dialects, as was discussed at length at a recent workshop at Uppsala University.^25^ Since, to my knowledge, deaf Tibetans in Amdo or Kham have yet to adopt some form of TibSL, or to develop other *deaf community sign languages* (Meir et al. 2012: 2), TibSL’s influence on other regional or local signing practices there seems, at present, a remote possibility.

Compared to many of the other 38 to 52 minority languages currently spoken in pockets within the Chinese Tibetosphere (Lewis et al. 2015a; Caixiangduojie 2014; Roche 2017), TibSL – when looked at through the UNESCO model – seems to be faring better in some respects, if not in number of absolute users. TibSL has received longer-term and greater NGO financial and logistical aid than others, and even some governmental recognition and support. The results have been promising and several major achievements for the language and rights of deaf Tibetans have been made between 2001 and 2015. These range from the intermittent, part-inclusion of TibSL classes for several years at the Lhasa Special School to the official recognition of Tibetan sign as China’s first “minority sign language” and several important TAR-wide policies and statements that have been issued since 2008.

These developments were in part a result of the TibSL language materials, such tangible “proofs” of a language being generally known to have vital symbolic force as well as political traction in legitimating (minority) languages. Also important to acknowledge are China’s commitments to international legal frameworks, such as the UNCRPD (cf. Haualand and Allen 2009, UN. 2006), as well as to national laws, including the National Law for the Protection of Persons with Disabilities (passed in 2008), and the Regulations on the Construction of Barrier-free Environments (from 2012). In conjunction with a rise in awareness of disability issues and rights among deaf Tibetans themselves, there has been an increased self-confidence and also a new sense of belonging among a number of deaf Tibetans in Lhasa. Many TibSL signers have a decidedly positive attitude towards their language, despite the sometimes great hurdles encountered due to a plethora of political sensitivities specific to Lhasa and the TAR, as well as societal prejudices vis-à-vis deaf people and sign language. Despite the many impressive gains made through the TibSL project, and the work of the TDA in collaboration with TDPF and HI, my preliminary assessment of the language through the UNESCO and the adapted iSLanDS model indicates that the language is still in a precarious situation. In UNESCO and iSLanDS terminology it would be judged as between definitely and severely endangered, hence warranting both language documentation and revitalisation. This would best be achieved through domain expansion, bi-lingual education in the TAR’s Special Schools, as well as the actual implementation of governmental policies in support of sign interpretation.

A final point for more in-depth consideration in any future work is the interrelationship between the revival of Tibetan that has taken place since 2008/2010 and the situation of TibSL in Lhasa. While the ‘pure Tibetan’ movement has had an often discouraging effect on many Tibetan users of minority languages (that is, speakers of non-major Tibetan dialects or languages), there seems to be a positive (and potentially mutually-supportive) association between those who promote the Tibetan language and those who promote TibSL. Deaf Tibetans have in many ways been excluded from full participation in Tibetan society for so long. The use of TibSL and the potential it opens up for the acquisition of written (and sometimes) spoken Tibetan has created a new sense, not just of belonging to a deaf community in Lhasa, but also to Tibetan society more broadly.

Robin has drawn our attention to the various Tibetan language initiatives and forums in response to threats to the teaching and use of Tibetan. These include for example literature, poems and blogs in praise of the Tibetan language (Robin 2014). Having translated and analysed several poems glorifying the beauty of the Tibetan alphabet, such as “*Ka, kha, ga, nga*, my life force”, “I am the Tibetan alphabet” and “Calling the alphabet from afar” (Robin 2014: 217– 220), she suggests that they “anthropomorphize the Tibetan alphabet, equating it with human body parts, in other words, with essential physical and mental components of Tibetans themselves” (Robin 2014: 217). This theme has also been taken up in a recent Tibetan blog post, showing images of the Tibetan consonants “being danced” (*gsal byed sum cu*’*i gar stabs*) by a playful Tibetan character (Tashi Norbu 2015). These poems and artwork allegorically suggest an intimate embodiment of Tibetan writing and of the Tibetan language. For those who finger spell the Tibetan alphabet, the embodiment of the Tibetan script is even more literal and “real”. The body becomes text and Tibetan text embodied. Perhaps this is an instance where the Tibetan script truly [104523] becomes the life force, the blood and soul of Tibetans, and TibSL signers at last integrated into pan-Tibetan aspirations.

## Figures and Tables

**Table 1:** UNESCO and iSLanDS criteria for the assessment of linguistic vitality and endangerment and the TibSL scores.

	UNESCO domains - assessing spoken languages	iSLanDS domains - adapted for assessing sign languages	Score UNESCO and ISLANDS	Additional ISLANDS domain score	Score description
1.	Intergenerational language transmission	Generational/age group use [for emerging sign languages]	4		unsafe/vulnerable
2.	Absolute numbers of speakers	Number of sign language users	small		
3.	Relative proportion of speakers within the total population	Proportion of signers within the reference community	1		Critically endangered
4.	Shifts in domains of language use	Domains of language use	4		unsafe/vulnerable
5.	Response to new domains and media	New domains, i.e. new media, including broadcast media and the internet	2		Severely endangered
6.	Availability of materials for language education and literacy	Materials for language spread and education	3		Definitely endangered
7.	Governmental and Institutional Language Attitudes and Policies, including Official Status and Use	Governmental and institutional language attitudes and policies, including official status and use	4		unsafe/vulnerable
		Use of the target sign language in deaf education		2	Severely endangered
8.	Community members’ attitudes	Reference community members’ attitudes towards their own sign language	2		Severely endangered
9.	Type and quality of documentation	Type and quality of documentation	2		
	-	Status of language programs		2	Severely endangered
			2,75	2,6	Between definitely and severely endangered

## Footnotes

1 I here use the term “deaf” to refer to all kinds of deaf people. A Deaf Studies convention is to use “deaf” to denote physiological deafness, “Deaf” to highlight cultural identity and “d/Deaf” for the often inherently mixed nature of the two (Senghas and Monaghan 2002). For readability’s sake these distinctions have been omitted here and it remains to be seen if such conceptual divisions are valid for the situation in Tibet.

2 The TAR is one of five provinces of the PRC hosting Tibetan populations. This article only deals with the situation in Lhasa and to some extent the TAR. To my knowledge there has not yet been any study of deaf Tibetans in the other provinces.

3 I have since been able to carry out more fieldwork but findings from this could not be included due to submission of this article in late 2015.

4 Government figures on the TAR and Lhasa population and particularly the percentage of Tibetans and Han Chinese among them are a notoriously political subject (cf. TIN 2003; Yeh and Henderson 2008).

5 The 2000 census states the total population of TAR was 2.6 million, with 94% of Tibetan ethnicity (TIN 2003). According to the TDA there were about 45,000–50,000 deaf in the whole of the TAR (2011). As the WHO-estimated average world-wide is that about 1 in 1000 is deaf or hard of hearing, there seems to be a higher than average occurrence of deafness in the TAR.

6 The figure is likely higher than that as many deaf I got to know had moved to the city due to better employment prospects and the possibility of meeting other deaf more easily. According to the 2006 Disability Census, as summarized in HI (2011: 6–7), the TAR is home to 194,000 people with some form of disability, and 26% of them are classified to have a “hearing or language disability” (*tingli can ji*), but there are no reliable Lhasa-specific figures available yet.

7 On homesigns more broadly, see Goldin-Meadow (2003) and Coppola et al. (2013).

8 In Tibetan, Bod ljongs ’on lkugs lhan tshogs. A direct English translation of this is the ‘Tibet Deaf and Mute Association’, but this was not the term used in English on the various publications and signs boards. The Tibetan name was officially changed to Bod ljongs ’on pa’i mthun tshogs (Tibetan Deaf Association) in 2011. The Chinese name of the association is Xi zang long ya xie hui.

9 For more details on the relations of TDPF, CDPF and the TDA shedding the now politically incorrect term “deaf and mute” due to its connotation of “deaf and dumb”, see Hofer and Sagli (2017).

10 Tib. Lha sa mig ser lob gra, Chin. Te shu tiao yu xue xiao jian jie.

11 Previously it was used only in internal HI documents and reports.

12 *Lkugs skad*, on the other hand, was the term used for spoken language that was not clear, or by someone who had problems speaking altogether.

13 The result of this would be that TibSL features on *Ethnologue* and other linguistic databases.

14 The standardisation of CSL shows similarities to the promotion of *Putonghua* since the 1950s, yet *Putonghua* was originally a western Beijing dialect, while CSL at least in its grammar was never used by deaf communities in China.

15 For discussion of international examples, see Manoghan et al. (2003) and for ASL, see Maher (1996).

16 Personal communication, Sonja Erlenkamp, December 2013.

17 This is now on the way, as part of my current research at the Universities of Bristol and Oxford.

18 The data presented here is also being contributed to the iSLanDS Sign Language Endangerment Research Project.

19 Thanks to Marjorie Unal for clarifying various official policies and the situation of TibSL teaching in the Lhasa school after 2008. She also alerted me to a combined CSL and TibSL curriculum developed by a professional Chinese sign expert for adult deaf Tibetans, published in 2010 in Chinese, Tibetan and with some of it translated into English. It follows a course of 13 units (each of three hours’ duration), “serving the purpose of literacy and learning sign language” (TDA 2010: 2).

20 There have been several one-month TibSL training courses for government teachers at the Special School (established in 2012), Shigatse which were officially requested by the directors and taught by the TDA activists. They were very successful in smoothing out what seemed an initially insurmountable communication divide that existed between the hearing Chinese (and some Tibetan) teachers and the exclusively deaf Tibetan children from rural areas, many of whom had had no or very little exposure to any Chinese before. It is unclear whether TibSL is currently used in some way in that deaf school or not. Given this current lack of information, and that this article refers only to the Lhasa-based TibSL reference community, these and future findings from outside of Lhasa will have to be taken into account in another assessment.

21 Future research is needed to establish the actual size and number of TibSL signers more precisely, for instance by following some of the methods suggested for such an endevour by Nonaka (2009).

22 A score of “3” in contrast is called “fair”, i. e. “there may be an adequate grammar, some dictionaries, and texts, but no everyday media; video recordings may exist in varying quality and with varying degree of annotation” (Zeshan et al. 2011: 11).

23 For instance in the case of *Khroskyabs* (or *Lavrung*) speakers in Jinchuan County as discussed in Lai Yunfan’s work, personal communication Gerald Roche, May 2015.

24 It is also worth noting that just because many TibSL users prefer TibSL over CSL, this does not mean they are uninterested in learning Chinese. A recent survey of twenty-one TDA members revealed that all of them wanted to increase their Chinese competence (HI 2011). This gives a clear indication that they, like hearing Tibetans and other minorities, need and want to learn and to improve their Chinese to better advance in society, especially in the job market.

25 The title of the workshop was ‘Minority Languages of the Chinese Tibetosphere: Ancient Trends, Contemporary Developments, and Future Prospects’, November 3–4, 2014, Hugo Valentin Centre, Uppsala University, Sweden.
